# Docetaxel, Cisplatin, and 5-fluorouracil (DCF) chemotherapy in the treatment of metastatic or unresectable locally recurrent anal squamous cell carcinoma: a phase II study of French interdisciplinary GERCOR and FFCD groups (Epitopes-HPV02 study)

**DOI:** 10.1186/s12885-017-3566-0

**Published:** 2017-08-25

**Authors:** Stefano Kim, Marine Jary, Thierry André, Véronique Vendrely, Bruno Buecher, Eric François, François-Clément Bidard, Sarah Dumont, Emmanuelle Samalin, Didier Peiffert, Simon Pernot, Nabil Baba-Hamed, Farid El Hajbi, Olivier Bouché, Jérôme Desrame, Aurélie Parzy, Mustapha Zoubir, Christophe Louvet, Jean-Baptiste Bachet, Thierry Nguyen, Meher Ben Abdelghani, Denis Smith, Christelle De La Fouchardière, Thomas Aparicio, Jaafar Bennouna, Jean-Marc Gornet, Marion Jacquin, Franck Bonnetain, Christophe Borg

**Affiliations:** 1 Centre Hospitalier Universitaire de Besançon, Besançon, France; 2 Clinical Investigational Center, CIC-1431, University Hospital of Besançon, Besançon, France; 3 INSERM, Unit 1098, University of Bourgogne Franche-Comté, Besançon, France; 4 Groupe Coopérateur Multidisciplinaire en Oncologie (GERCOR) Oncology Multidisciplinary Group, Besançon, France; 5 Fédération Francophone de Cancérologie Digestive (FFCD), Dijon, France; 6 Hôpital Saint Antoine, Paris, France; 7 Centre Hospitalier Universitaire de Bordeaux, Bordeaux, France; 8 Institut Curie, Paris, France; 9 Centre Antoine-Lacassagne, Nice, France; 10 Institut du Cancer de Montpellier, Montpellier, France; 11 Institut de Cancérologie de Lorraine, Nancy, France; 12 Hôpital européen Georges-Pompidou, Paris, France; 13 Groupe Hospitalier Paris Saint-Joseph, Paris, France; 14 Centre Oscar Lambret, Lille, France; 15 Centre Hospitalier Universitaire de Reims, Reims, France; 16 Hôpital privé Jean Mermoz, Lyon, France; 17 Centre François Baclesse, Caen, France; 18 Hôpital Privé des Peupliers, Paris, France; 19 Institut Mutualiste Montsouris, Paris, France; 20 Hôpital Pitié Salpêtrière, Paris, France; 21 Polyclique de Franche-Comté, Besançon, France; 22 Centre Paul Strauss, Strasbourg, France; 23 Centre Léon Bérard, Lyon, France; 24 Hôpital Avicenne, Bobigny, France; 25 Institut de Cancérologie de l’Ouest, Nantes, France; 26 Hôpital Saint Louis, Paris, France; 27 Cancéropôle Grand Est, Besançon, France; 28 Methodology and Quality of Life in Oncology Unit, University Hospital of Besançon, Besançon, France; 29 French National Platform Quality of Life and Cancer, Besançon, France; 30 Department of Oncology, Jean Minjoz University Teaching Hospital, 3 Boulevard Alexander Fleming, F-25030 Besancon, France

**Keywords:** Anal carcinoma, Advanced, Metastatic, Docetaxel, And chemotherapy

## Abstract

**Background:**

The squamous cell carcinoma of the anus (SCCA) is a rare disease, but its incidence is markedly increasing. About 15% of patients are diagnosed at metastatic stage, and more than 20% with a localized disease treated by chemoradiotherapy (CRT) will recur. In advanced SCCA, cisplatin and 5-fluorouracil (CF) combination is the standard option but complete response is a rare event and the prognosis remains poor with most disease progression occurring within the first 12 months.

We have previously published the potential role of the addition of docetaxel (D). Among 8 consecutive patients with advanced recurrent SCCA after CRT, the DCF regimen induced a complete response in 4 patients, including 3 pathological complete responses.

Then, the Epitopes-HPV02 study was designed to confirm the interest of DCF regimen in SCCA patients.

**Methods:**

This multicentre phase II trial assesses the DCF regimen in advanced SCCA patients. Main eligibility criteria are: histologically proven SCCA, unresectable locally advanced recurrent or metastatic disease, Eastern Cooperative Oncology Group-performance status (ECOG-PS) <2, and being eligible for DCF. Patients receive either 6 cycles of standard DCF or 8 cycles of modified DCF depending on age (> vs. ≤ 75 years-old) and ECOG-PS (0 vs. 1). The trial was set up based on a Simon’s optimal two-stage design for phase II trials, allowing an early futility interim analysis. The primary endpoint is the observed progression-free survival (PFS) rate at 12 months from the first DCF cycle. A PFS rate below 10% is considered uninteresting, while a PFS rate above 25% is expected. With a unilateral alpha error of 5% and a statistical power of 90%, 66 evaluable patients should be included. Main secondary endpoints are overall survival, PFS, response rate, safety, health-related quality of life, and the correlation of biomarkers with treatment efficacy.

**Discussion:**

Since the recommended CF regimen is based in a small retrospective analysis and generates a low rate of complete responses, the Epitopes-HPV02 study will establish a new standard in case of a positive result. Associated biomarker studies will contribute to understand the underlying mechanism of resistance and the role of immunity in SCCA.

**Trial registration:**

NCT02402842, EudraCT: 2014–001789-81.

## Background

Even if squamous cell carcinoma of the anal canal (SCCA) is a rare disease, representing only 1%–5% of all gastrointestinal malignancies, its incidence is steadily increasing among men as well as women [1–3]. This increase is likely related to the higher prevalence of the anal human papilloma virus (HPV) infection, since HPV-related oncoproteins (E6 and E7) are indeed expressed in more than 90% of SCCA [4].

The great majority of SCCA patients are diagnosed at a localized stage, and the standard treatment at this stage consists in the concurrent chemoradiation with mitomycin C (MMC) plus 5-fluorouracil (5FU) [1, 5, 6]. Nevertheless, more than 20% of patients will develop locally advanced recurrences or metastases, and this recurrence rate can reach 50% in high burden tumours (>5 cm) or in case of lymph node involvement [7–10].

A salvage surgery might be proposed to patients in case of resectable local progression. When surgery is not possible, the treatment relies on systemic chemotherapy. However, evidence to define the appropriate chemotherapy regimen is lacking [11, 12]. The combination of CDDP and 5FU is recommended for advanced SCCA, based on a retrospective analysis of 19 patients, as illustrated by the recently revised guidelines of the National Comprehensive Cancer Network (version 2.2016), which acknowledged that no other regimen has shown to be effective. However, complete remission is observed in less than 5% of cases, and the great majority of patients have disease progression before 12 months [13, 14].

The absence of a curative therapeutic option available to treat relapsing patients led to the initiation of clinical trials in order to intensify preoperative CRT and decrease primary treatment failure. ACCORD03 and RTOG 98–11 trials were then conducted to evaluate the interest of CDDP and 5FU chemotherapy as an induction treatment and in combination with radiotherapy, compared to standard CRT with MMC and 5FU. Nevertheless, these studies failed to improve local and distant recurrence rates [7, 15]. Thus, the overall outcome for patients with non-resectable relapses or metastatic disease remain dismal with an overall 5-year survival rate below 20% for stage IV disease, and the development of effective salvage systemic therapies is still a relevant issue for these patients.

Docetaxel is a potent microtubule-stabilizing agent with an antitumor activity leading to mitosis arrest and cell death. It has been previously proposed that a loss of normal p53 function confers sensitization to taxane chemotherapy by increasing G2/M arrest and apoptosis [16]. Since the association between anal carcinoma and human papilloma virus (HPV) infection is especially strong and the E6 oncoprotein encoded by HPV types 16 and 18 induces the degradation of p53, we hypothesized that SCCA might be sensitive to taxane-containing chemotherapies [4, 17]. In addition, docetaxel has previously shown to increase endoplasmic reticulum stress and to induce an immunogenic cell death of cancer cells [18].

The improvement of the overall survival without any impact on toxic death through the addition of docetaxel to CDDP plus 5FU (DCF) has already been shown in advanced squamous cell carcinoma of the head and neck [19]. Then, considering the poor outcome with CDDP and 5FU, we decided to treat metastatic and unresectable local recurrent SCCA patients using the DCF regimen. Our result on the first 8 consecutive patients was previously published [20]. This regimen triggered an unexpected high level of long-term complete remission in 4 patients, 3 of them with complete pathological responses of their metastases. Three of these patients are still in complete remission after 73, 65, and 48 months of follow-up. The other patient died due to a sepsis complication of CRT-induced chronic pelvis infection, following 118 months of survival from the first DCF cycle, and without disease recurrence at the time of death. Then, we performed a translational research study. Interestingly, a p16+ and p53− phenotype was observed in all patients in remission. Furthermore, high levels of specific T cell responses against HPV16-derived E6/E7 and telomerase were detected in 50% of complete responders, suggesting the potential restoration of cancer immunosurveillance by this regimen [21].

Therefore, our preliminary results suggested a new potential role of taxane-based chemotherapy in SCCA patients. Then, a French network including 25 centers was organized with collaborative groups (Groupe Coopérateur Multidisciplinaire en Oncologie (GERCOR) Oncology Multidisciplinary Group and Fédération Francophone de Cancérologie Digestive (FFCD)) to conduct a multicentric prospective clinical trial to validate the role of DCF regimen in SCCA.

Our scientific committee has decided to perform a single arm study to validate our previous encouraging results of the DCF regimen in unresectable locally recurrent or metastatic SCCA patients. Indeed, no randomized clinical trials are currently available to determine a standard of care for relapsing or metastatic SCCA patients, and the combination of cisplatin-5FU only demonstrated a modest activity in a small retrospective analysis, with progression-disease before 12 months in the great majority of the patients [13, 14].

In addition, ancillary biomarker studies in tissues and plasma will also be performed in the Epitopes-HPV02 trial. Considering the specific tumorigenesis of SCCA patients, it will provide a unique opportunity to find out the underlying mechanism of resistance and response, including the tumour immunity, in a dynamic manner.

## Methods and analysis

The Epitopes-HPV02 study is a multicentre, single-arm phase II study. It was developed by the “National Institute of Health and Medical Research (INSERM), Unit 1098” and “Clinical Investigational Center (CIC) 1431”, and is supported by GERCOR and FFCD collaborative oncologic groups. The study is coordinated by the “Department of Clinical Research and Innovation” of University Hospital of Besançon. The data management is undertaken by the “Methodology and Quality of Life Unit in Oncology”* of the University Hospital of Besançon, and the trial is registered on the clinicaltrials.gov database (NCT02402842), partly funded by a research grant from the University Hospital of Besançon, and conducted in accordance with the Declaration of Helsinki and the Good Clinical Practice.

* http://www.umqvc.org/en/index.html

### Study objectives

*The primary end-point* is to evaluate the observed progression-free survival rate at 12 months from the initiation of DCF in patients with unresectable locally advanced recurrent, or metastatic SCCA.

*The secondary end-points* are:

- To evaluate the overall survival
- To evaluate the progression-free survival
- To evaluate the objective response rate
- To assess the safety of DCF in advanced SCCA
- To assess the health-related quality of life (QoL)
- To evaluate HPV and telomerase-specific T cell responses before and after DCF treatment, and to correlate them to the survival
- To analyze the tumor genotyping for HPV, p53, neoantigens. The correlation of these biomarkers with treatment efficacy
- To investigate the prognostic value of tumor-infiltrating lymphocytes

### Patient selection

The study population consists of patients with SCCA at metastatic stage or with locally advanced recurrence after CRT, non-eligible for salvage surgery due to the extension of the disease. Patients should be eligible for DCF, with a performance status ECOG-PS 0 or 1, and with adequate organ function. The local institutional multidisciplinary board of each center will identify eligible patients. The inclusion and exclusion criteria are listed in Table 1.

**Table 1:** Inclusion and exclusion criteria of the trial

*Inclusion criteria*
➢ Histologically proved, and unresectable locally advanced recurrent or metastatic SCCA patient
➢ Eligible for DCF or mDCF regimen
➢ Age ≥ 18 years
➢ ECOG-PS of 0 or 1
➢ Signed written informed consent
*Exclusion Criteria:*
➢ Known hypersensitivity or contraindication to any of the study drugs (docetaxel, cisplatin, 5-fluorouracil).
➢ Previous chemotherapy for metastatic disease
➢ Previous chemotherapy by paclitaxel, docetaxel or navelbine
➢ Previous chemotherapy by cisplatin, except of concomitant radiotherapy
➢ HIV positive patient with lymphocyte CD4 count under 400/mm^3^
➢ Concomitant treatment with a CYP3A4 inhibitor*
➢ Inadequate organ function
➢ Other malignancy within the last 3 years, except for adequately treated carcinoma in situ of the cervix or squamous carcinoma of the skin, or adequately controlled limited basal cell skin cancer.
➢ Simultaneous participation in another clinical study
➢ Pregnancy, breast-feeding or absence of adequate contraception for fertile patients
➢ Patients with any disabling medical or psychiatric condition or disease for this study.
➢ Presence of peripheral neuropathy
➢ Presence of auditory disorders
➢ Yellow fever vaccination, prophylactic use of phenytoin, live-attenuated vaccines
➢ Inadequate bone marrow, renal and liver function
○ Neutrophil count <1500/mm^3^,
○ Platelet count <100,000/mm^3^,
○ Clearance of creatinine (Cockcroft formulae) < 60 ml/min,
○ AST and ALT >2.5 × upper limit of normal (> 5 x upper limit of normal in case of known liver metastases)
○ Total bilirubin >2.5 × upper limit of normal
* The treatment can be replaced or stopped before inclusion

### DCF treatment

Patients will receive 6 cycles of DCF regimen (docetaxel 75 mg/m^2^ day, CDDP 75 mg/m^2^ and 5FU at 750 mg/m^2^/day for 5 days) every 3 weeks or 8 cycles of modified-DCF regimen (mDCF, docetaxel 40 mg/m^2^ day, CDDP 40 mg/m^2^ day and 5-FU at 1200 mg/m^2^/day for 2 days) every 2 weeks, according to their clinical status. The standard DCF regimen is recommended for patients up to 75 years and with ECOG-PS of 0, whereas the mDCF is recommended for those >75 years-old or ECOG-PS of 1.

Granulocyte colony-stimulating factor will be systematically administered as primary prophylaxis of febrile neutropenia for both regimens.

### Post per-protocol treatment

At the end of planned DCF cycles, complementary local treatments like surgery of metastases or radiotherapy will be allowed at the discretion of each local institutional multidisciplinary board’s decision.

### Evaluation, laboratory tests and follow-up

CT scans are planned at baseline, after 3rd and 6th cycles of DCF regimen (or after 4th and 8th cycles of modified-DCF regimen) and then every three months until disease progression, or death. Tumour assessment will be carried out according to RECIST criteria version 1.1. A PET scan will be performed before and at the end of chemotherapies.

The blood samples for immuno-monitoring will be performed before DCF and at first follow-up visit after the last DCF cycle.

The QoL will be measured by the EORTC QLQ-C30 questionnaire at inclusion, and every 2 cycles of chemotherapy.

Patients will be followed-up every 3 months, up to 3 years from enrolment, or death. A CT-scan, blood analysis, clinical examination, and QoL measurement will be performed at each follow-up visit Table 2.

**Table 2:** time and events table

	Screening & Inclusion		Treatment			End of treatment visit	Follow-up visit
	≤28 days	≤7 days	3 DCF or 4 mDCF cycles	Mid-treatment assessment	3 DCF or 4 mDCF cycles	28 days after last cycle	3 monthly for 3 years or death
DCF/mDCF treatment			♦^a^		♦^a^		
Informed consent	♦						
Eligibility criteria	♦						
History and concomitant treatments	♦						
Physical assessment including weight, height, ECOG-PS		♦					
Vital signs including pulse & blood pressure		♦					
12-lead ECG		♦					
Pregnancy test		♦					
Standard blood test^b^		♦					
Tumor tissue recovery	♦						
Chest, abdomen and pelvis CT scan	♦			♦		♦	♦
FDG-PET-CT^c^	♦					♦	
QoL assessment^d^		♦	♦		♦	♦	♦
Blood test for translational study		♦				♦	
Toxicity/compliance assessment			♦		♦	♦	
Vital & progression status, late toxicity recovery							♦

^a^3 cycles of 3 weekly DCF (docetaxel, cisplatin, 5FU) or 4 cycles of biweekly modified DCF

^b^complete blood count, sodium, potassium, creatinine, bilirubin, alkaline phosphatase, alanine aminotransferase, aspartate aminotransferase, glucose, albumin, calcium, magnesium

^c^fluorodeoxyglucose-positron emission tomography

^d^EORTC-QLQ-C30 & development of a specific anal module by EORTC QoL group

Abbreviations: ECOG-PS, Eastern Cooperative Oncology Group-performance status; ECG, electrocardiogram; FDG-PET-CT, fluorodeoxyglucose-positron emission tomography–computed tomography; QoL, health-related quality of life

### Data management

For each patient enrolled in the study, the investigators must document all required data in the corresponding source documents. These data must then be entered in electronic case report form (eCRF), which will be accessible only by authorized persons via secured web connection. One eCRF will be created for each patient. The investigator has the responsibility for its completion, proof reading, as well as its approval after the final verification for the authenticity and accuracy of all entered data. Once completed, eCRF will be locked and monitored. The promoter will assure data quality control. A clinical research assistant mandated by the promoter will perform physical visits at each participating center to control all entered data, to reassure the good clinical practice, and to respect all legal responsibilities.

### Statistical considerations

Epitopes-HPV02 is a multicentre, open-label, singe-arm, phase 2 trial, designed with Simon’s optimal two-stage method [22], and undertaken in 25 cancer centres in France. This method schedules an early stop at first stage in case of futility (inefficacity of the DCF regimen). The primary objective is the observed PFS rate at 12th month from the first DCF cycle. PFS rate of 25% is expected, and 10% is considered uninteresting. Using the Simon’s method, with a power of 90% and one-sided α of 0.05, 66 patients will be enrolled for final analysis.

#### At stage 1

After the first 21 patients are enrolled with at least 12 months of follow-up, the trial will be stopped if we observe less than 3 alive patients without progression, and we will declare the treatment as uninteresting. On the contrary, the trial will be pursuit if we observe 3 or more alive patients without progression at 12 months, and 45 additional patients will be included.

#### At stage 2

After 66 patients enrolled, the observation of less than 11 alive patients without progression at 12 months will conclude the study as negative. Otherwise, 11 or more alive patients without progression at 12 months will conclude the study as positive, and the DCF treatment as clinically interesting.

### Primary analysis

A PFS rate superior to 25% at 12 months from the first DCF cycle is defined as primary end-point. PFS is defined as the time from the first DCF to progression or death from any cause, evaluated by RECIST criteria version 1.1.

### Secondary analyses

- Overall survival, defined as the time from the date of the first DCF cycle to death from any cause.
- Objective response rate evaluated by RECIST criteria, v1.1. All CT scans and PET scans will be centralized for a blinded radiological assessment.
- Toxicities graded according to the National Cancer Institute - Common Terminology Criteria for Adverse Events (NCI-CTCAE) criteria, version 4.03
- QoL analysis measured by the EORTC-QLQ-C30 & time to QoL score deterioration with a minimal clinically important difference of 10 points. Patients also participate to an ancillary study for the development of a specific anal module with EORTC QoL group.
- HPV and telomerase-specific T cell responses before and after treatment measured by ELISPOT assay, as previously described by our team [23, 24].
- Characterization of tumour genotyping for HPV, p53, and neoantigens using the next-generation sequencing.
- Tumour-infiltrating lymphocytes analysis (TIL isolation or immunohistochemical analysis of Tbet, CD8, Foxp3, RoR-γt).

The statistical analysis will be performed with the modified intention to treat population (mITT, population with at least one cycle of DCF/mDCF regimen).

Since this is a non-comparative study, the statistical analysis will be mostly descriptive. For efficacy analysis, a 95% confidence interval (95% CI) will be performed. For the exploratory construction of the predictive model of overall survival, 10% will be considered as the threshold of significance for the pre-selection of variables, and 5% for the construction of multivariate analysis.

The qualitative variables will be described by usual statistics: effective and percentage (in relation to the included effective) of each modality. The quantitative variables will be described by the median, mean, standard deviation, the extreme values and interquartile ranges. For each variable, missing data will be described, and 95% CI will be reported.

The median progression-free survival (PFS) and overall survival (OS), with its 95% CI will be determined by Kaplan-Meier method. PFS will be calculated between the inclusion date and the date of the first progression (local, regional, metastatic, second cancer) or the last follow-up date for alive patients without progression, and the OS will be calculated between the inclusion date and the date of death. The median follow-up will be calculated by reverse Kaplan Meier method. Exploratory investigations will be undertaken to identify the independent factor related to PFS (age, ECOG-PS, type of metastases, response to prior CRT, QoL…) using univariate and multivariate Cox models. The correlation between the possible variables related to the event will be determined (correlation of rang, test of chi^2^ or ANOVA depending on types of variables) before the construction of multivariate models. In case of several variables strongly correlated, only the variable displaying the higher correlation with PFS will be retained.

QoL analysis:

All analysis will be performed in a mITT (population with at least one QoL evaluation performed).

After the generation of the scores following the EORTC recommendations, the scores will be described at each time of measure using median (Min-Max) and mean (standard deviation).

Missing data and random property of these data will be studied. The analysis of the random property of missing data will be done by comparison of those patients who completed whole questionnaires at inclusion versus those patients with at least one missing data. This comparison will be performed over the complete clinical and socio-demographic data recovered at inclusion. In case of a possible confounding factor, a multiple imputation of scores could be performed in the analysis of sensitivity taking into account these factors. A longitudinal analysis of the time until definitive deterioration of a score and the survival without deterioration of a score, will be conducted using the Kaplan-Meier method and described by median with its 95% CI. The time to deterioration of a score of QoL will be defined as the interval of time between the date of enrolment and the appearance of at least 10 points deterioration of the QoL score compared to the QoL score at baseline, without later improvement of more than 10 points compared to the QoL score at baseline, or death [25]. As an exploratory manner, univariate and multivariate Cox regression models for the 5 targeted-dimensions will be performed.

As part of sensitivity analysis, these analyses will be repeated using the “Inverse Probability of Treatment Weighting” technique of propensity score (by comparison of the profile of complete versus non-complete responders using the logistic regression model) and after the multiple imputations of the scores.

A difference of at least 10 points will be considered as the minimal clinically important difference (MCID). Analysis of sensitivity with a MCID of 5 points will be performed.

### End of trial

This study will end once all 66 recruited patients have completed 3 years of follow-up or have died, whichever comes first. Every patient participating in this study can stop the trial at any moment, and have no need to justify the reason for this decision. In that case, patients will pursue the standard medical care.

The reason for an early end of the study must be declared as on of the following criterion: patient’s decision, severe adverse event, protocol deviation, lost of follow-up, or death.

As above specified, the study will be stopped early after first-stage analysis, and declared uninteresting if less than 3 among the first 21 enrolled patients are progression-free after 12 months from the first DCF cycle.

### Monitoring and safety

A Data and Safety Monitoring Committee (DSMC) is implemented to safeguard participant safety. DSMC members are conformed by the study-independent pharmacovigilance department, methodologists, and the principal study investigator and coordinator.

This committee will perform meetings in case of potential safety signals in the study population detected by the pharmacovigilance department.

The information regarding adverse events (AEs) will be elicited at each contact with the participant, by appropriate questioning and examinations. All AEs will be declared if they occur between the signature of the consent form and 4 weeks after the last administration of the study treatment. AEs will also be recovered during the follow-up period if they are possibly related to the study, and at any time if they are possibly related to the study treatment.

The DSMC will perform an annual security report at each anniversary date from the study authorization delivery date by the French Health Products Safety Agency. This report will then be sent to the Committee for Protection of Persons in the 60 days following the authorization anniversary date.

## Discussion

The recommended cisplatin and 5FU chemotherapy generates a low rate of CRs in advanced SCCA. Moreover, cisplatin–5FU combination failed to improve the clinical outcomes of non-previously pre-treated SCCA patients in two randomized clinical trials [7, 8]. The addition of docetaxel to cisplatin-5FU demonstrated an encouraging 50% of complete response rate suggesting a particular mechanism of taxanes to induce complete remission in advanced SCCA [20]. The DCF regimen was also effective in radiation-resistant SCCA, since 2 among 4 complete responders had relapsed lesions in previously irradiated fields. These observations are in line with the clinical efficacy reported in advanced SCCA patients with paclitaxel, another potent microtubule-stabilizing agent. Paclitaxel in monotherapy has shown to be active in CDDP–5FU refractory SCCA patients [26]. In this study, seven patients were treated with weekly paclitaxel after failure of CDDP and 5FU. Three partial responses and 1 CR were observed. In another study, paclitaxel was used in five metastatic patients and induced three partial responses [27]. The potential interest of paclitaxel combined with 5FU and carboplatin was also assessed in advanced squamous cell carcinoma from several origins [28]. Seven SCCA patients were included in this phase II clinical trial. Two partial and two CRs were reported with long remissions. Altogether, including the patients reported in our previous study, 30 advanced or metastatic SCCA patients were treated with a taxane-based chemotherapy. Seven of these patients (23.3%) treated in different institutions achieved either a CR or a long-lasting complete remission [20]. Then, our primary objective in Epitopes-HPV02 trial is the long-lasting response rate with DCF regimen, defined as progression-free at 12 months from the first DCF cycle, in ≥25% of patients. Our redaction committee has preferred PFS instead of CR since the response assessment by RECIST criteria could be difficult to perform in recurrent lesion on irradiated field, and because most progressions occur before 12 months in case of absence of CR.

### Perspectives

Since 1996, no significant progress has been achieved in SCCA patients. The Epitopes-HPV02 study will establish a new standard in advanced SCCA patients in case of a positive result. Associated biomarker studies will provide an important opportunity to understand the underlying mechanism of resistance as well as the role of immunity associated with SCCA, which is mostly induced by HPV infection with a particular tumorigenesis.

Our trial could also provide the rational for immunotherapy and its combinations with chemo and radiotherapy in SCCA patients.

## Abbreviations

5FU: 5-fluorouracil
CDDP: Cisplatin
CF: Cisplatin and 5-fluorouracil
CR: Complete response
CRT: Chemoradiotherapy
D: Docetaxel
DCF: Docetaxel, cisplatin and 5-fluorouracil
ECOG-PS: Eastern Cooperative Oncology Group-performance status
HPV: Human papilloma virus
mITT: Modified intention to treat
MMC: Mitomycin C
Modified DCF: mDCF, docetaxel 40 mg/m^2^ day, CDDP 40 mg/m^2^ day and 5-FU at 1200 mg/m^2^/day for 2 days, every 2 weeks
OS: Overall survival
PFS: progression-free survival
QoL: Health-related quality of life
SCCA: Squamous cell carcinoma of the anus
Standard DCF: DCF, docetaxel 75 mg/m^2^ day, CDDP 75 mg/m^2^ and 5FU at 750 mg/m^2^/day for 5 days, every 3 weeks
